# Implantation of an attachment tube preserves knee extension after nonunion of Felix IV fracture: a case report

**DOI:** 10.1186/s13256-021-03095-8

**Published:** 2021-10-22

**Authors:** Alena Richter, Henning Windhagen, Max Ettinger

**Affiliations:** Orthopaedic Department – DIAKOVERE Annastift, Hannover Medical School, Anna-Von-Borries Str. 1-7, 30625 Hannover, Germany

**Keywords:** Case report, Felix IV fracture, Periprosthetic fracture, Attachment-tube, Knee joint extension

## Abstract

**Background:**

While commonly utilized to fix tissue and muscles to megaprostheses to restore function and stability after tumor surgery, an attachment tube was used as a synthetic reconstruction of the knee joint’s extension mechanism after nonunion of Felix IV C fracture. Fixation of the tibial fragment, and therefore its osteointegration, is complicated after total knee arthroplasty, causing tibial tubercle dislocation.

**Case presentation:**

A 61-year-old German patient presented to our clinic with Felix IV C fracture, persistent knee pain, and reduced knee extension strength. In this special case, mobilization and reattachment of the tibial tubercle was not possible because of necrosis and underlying tibial component. Therefore, we covered the defect with cement and used an polyethylene terephthalate tube for knee extension system augmentation. Follow-up after 10 months demonstrated a good clinical result.

**Conclusion:**

The management of Felix IV C fractures is complicated by the underlying prosthesis resulting in redislocation of the fragment and persistent symptoms of pain and reduced functionality. We here present a new surgical technique to treat periprosthetic fracture complicated by tibial tubercle dislocation. Good clinical and radiologic results on follow-up after 10 months indicate the use of attachment tubes as a suitable surgical technique to restore knee joint extension and to reduce knee pain after dislocated Felix IV C fracture.

## Background

Periprosthetic fractures of the tibia have a low prevalence of 0.4–1.7% [1], but since there the number of total knee arthroplasties (TKA) is increasing, it has become a significant complication in orthopedic surgery [2]. Therapy consists of osteosynthesis with plates or screws, but since the tibial stems limit the options of fragment refixation, periprosthetic tibial fractures are difficult to treat, having a high risk of nonunion and dislocation [3].

Herein, we report the reconstruction of the knee joint’s extension system with a polyethylene terephthalate tube after nonunion of tibial Felix IV C fracture [4].

## Case presentation

**Fig. 1:**
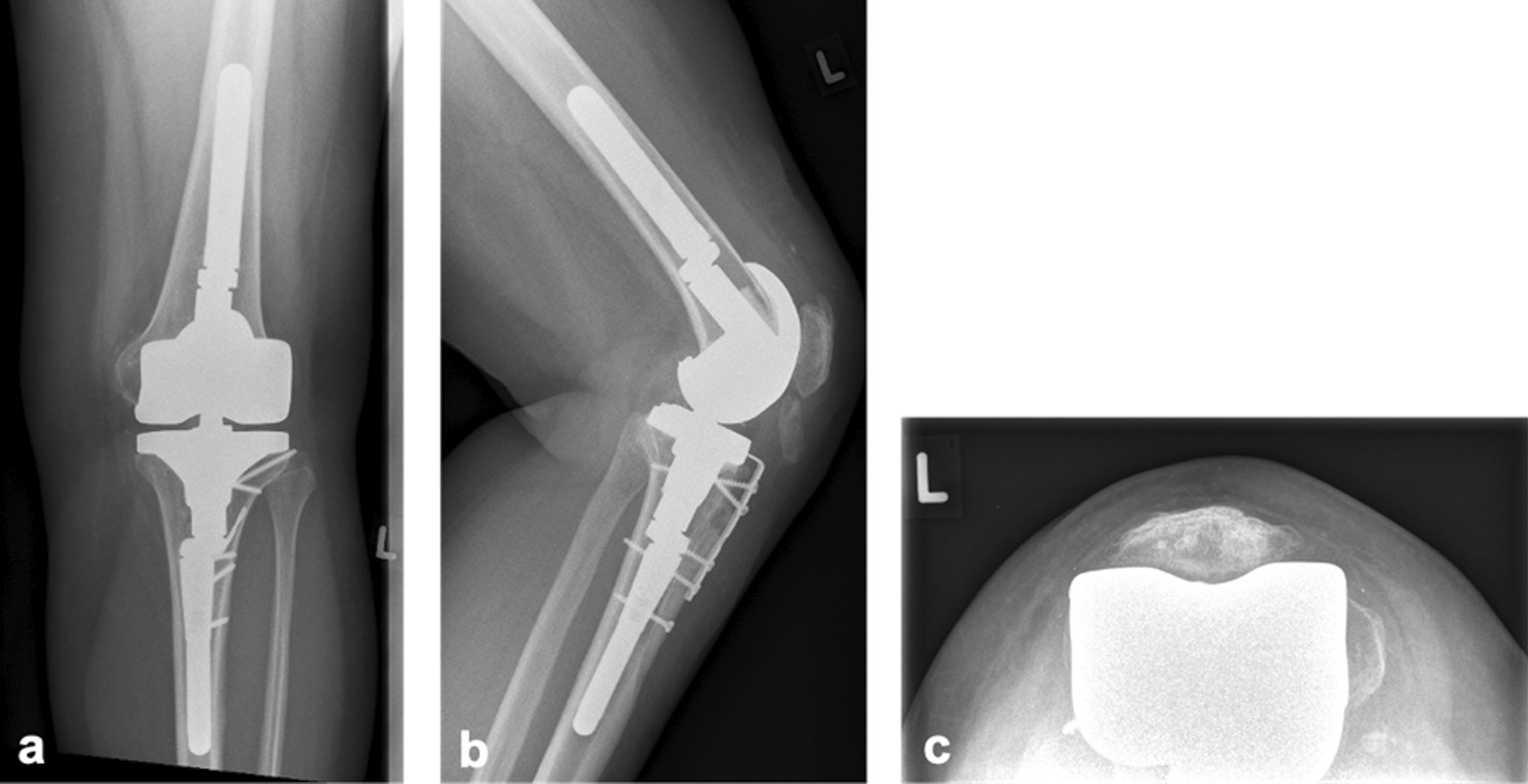
Preoperative x-ray shows dislocated tibial tubercle, one-third plate, rotation hinge prosthesis without loosening signs and regular patella tracking.** a** Frontal view.** b** Sagittal view shows dislocated tibial tubercle.** c** Axial view shows regular patella tracking

A 61-year-old German woman presented to our orthopedic clinic after Felix IV fracture in revision total knee arthroplasty. Two years ago, the patient had undergone revision TKA with a rotating hinge prosthesis complicated by an intraoperative fracture of the tibial tubercle. Screw fixation had been performed, but 3 months later nonunion of the fragment had persisted, resulting in plate osteosynthesis with a one-third tubular plate. After another dislocation of the tibial tubercle, the patient presented to our clinic with anterior knee pain, loss of extension strength, and a feeling of rotational instability. On clinical examination, pressure pain of the proximal tibia, decreased extension strength to Janda 3/5, and inability to raise the extended knee were noticed. This maintained extensor function is the result of an intact medial and lateral retinaculum. The knee’s range of motion was 0–110° of flexion with preserved mediolateral and anterior/posterior stability. X-ray showed the rotation hinge prosthesis without loosening signs, the one-third tubular plate, and the dislocated tibial tubercle fragment indicating persistent Felix IV C fracture (Fig. 1).

**Fig. 2:**
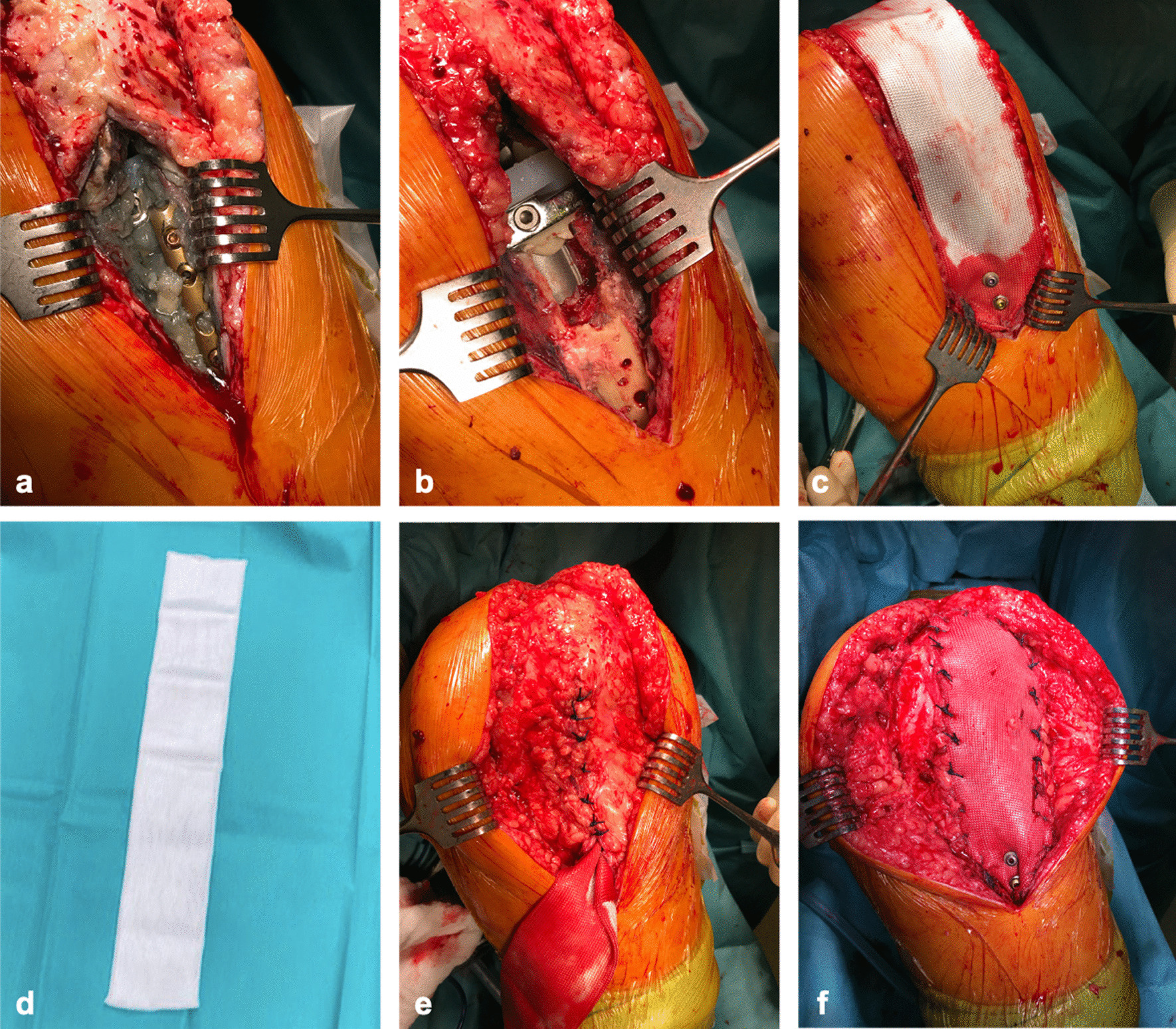
Intraoperative procedures:** a** After preparation of subcutaneous tissue, the one-third plate appeared completely surrounded with metallosis.** b** The necrotic tibial tubercle was removed so that the tibial prosthesis was exposed.** c**,** d** The polyethylene terephthalate tube was doubled to form a planar sheet and fixed with two cancellous bone screws in the proximal tibia.** e** Joint capsule was sutured so that the attachment-tube is arranged above.** f** The attachment-tube was fixed with non absorbable ORTHOCORD® at the joint capsule and the M. quadriceps femoris

**Fig. 3:**
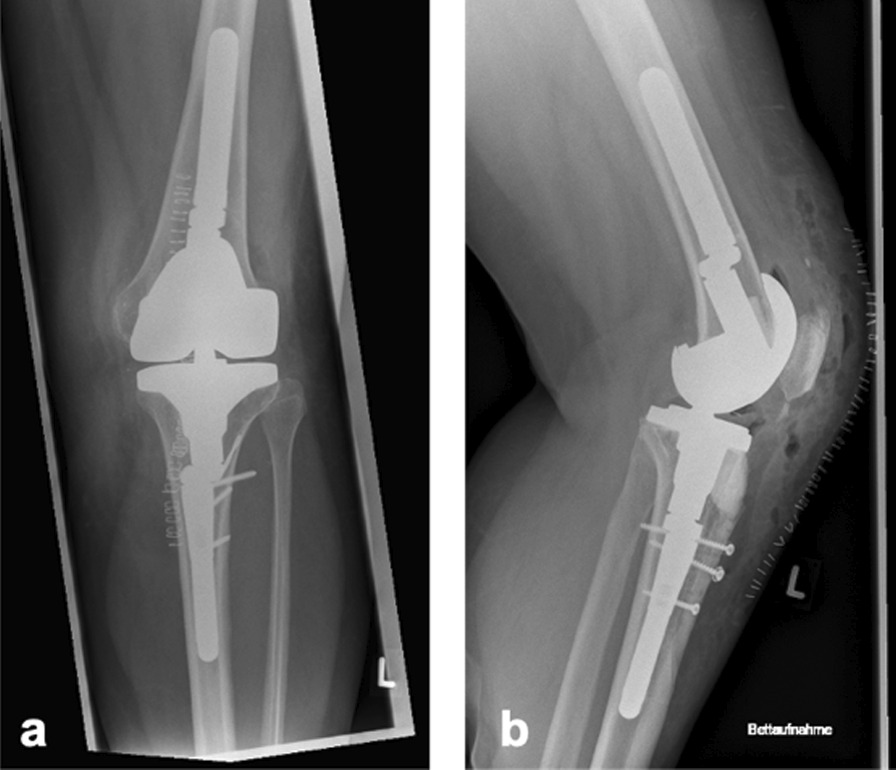
Postoperative x-ray shows cement augmentation of resected tibial tubercle and regular patella position.** a** Frontal view.** b** Sagittal view shows cement augmentation and correct patella position without cranial dislocation

Reconstruction of the knee joint’s extension system was planned. After preparation of the subcutaneous tissue, the dislocated tibial tubercle appeared surrounded by extensive metallosis (Fig. 2a) presumably induced by the contact between the one-third tubular plate and the tibial prosthesis. Following the removal of the plate, the fragment proved to be necrotic requiring a total extirpation instead of mobilization and refixation. Subsequently, the ventral surface of the tibial prosthesis was exposed (Fig. 2b). Reconstruction of the extension system should be performed by the implantation of MUTARS attachment tube made of polyethylene terephthalate. Since the tibial prosthesis did not offer any connecting points for the synthetic graft, cement was used as an extender to simultaneously serve as fixing point and to preserve the prosthesis from loosening. Afterwards, the tube was doubled into a laminar sheet and fixed with two cancellous bone screws in the ventral tibia (Fig. 2c, d). ORTHOCORD sutures were used to attach the tube to the articular capsule still beyond the patella (Fig. 2d,e). Extensive jet lavage was performed before wound closure. Check of patellar tracking was promising; likewise, postoperative X-ray showed regular patella position (Fig. 3).

To support the integration of the tube in the surrounding subcutaneous tissue, knee flexion was at first limited to 30° and then escalated to 60° and 90° every 2 weeks with full weight-bearing.

**Fig. 4:**
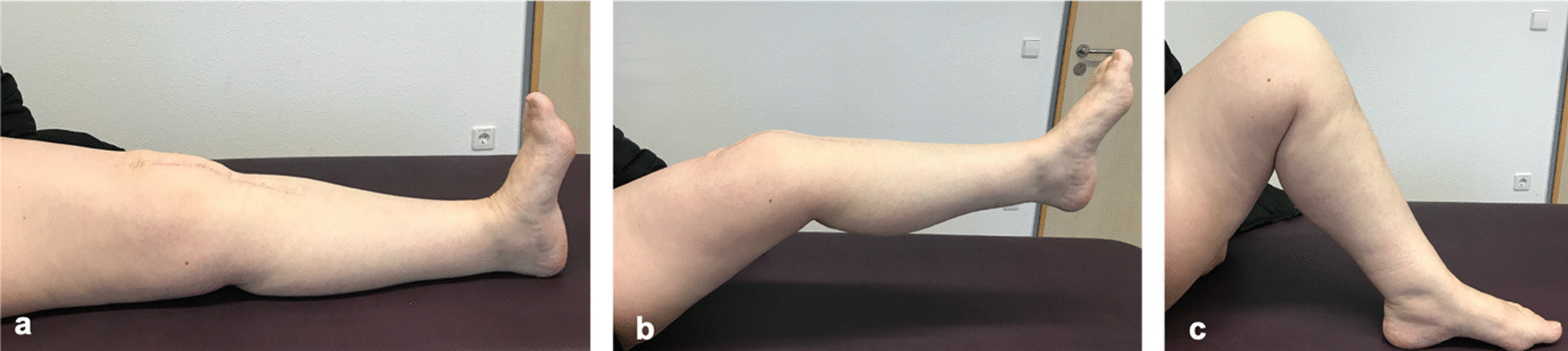
Clinical follow up after ten month: In the clinical examination the patient showed a range of motion with extension/flexion 0-0-110°, straight-leg-raise was completely possible.** a** Full knee extension of 0°.** b** Straight-leg-raise.** c** Knee flexion until 110°

**Fig. 5:**
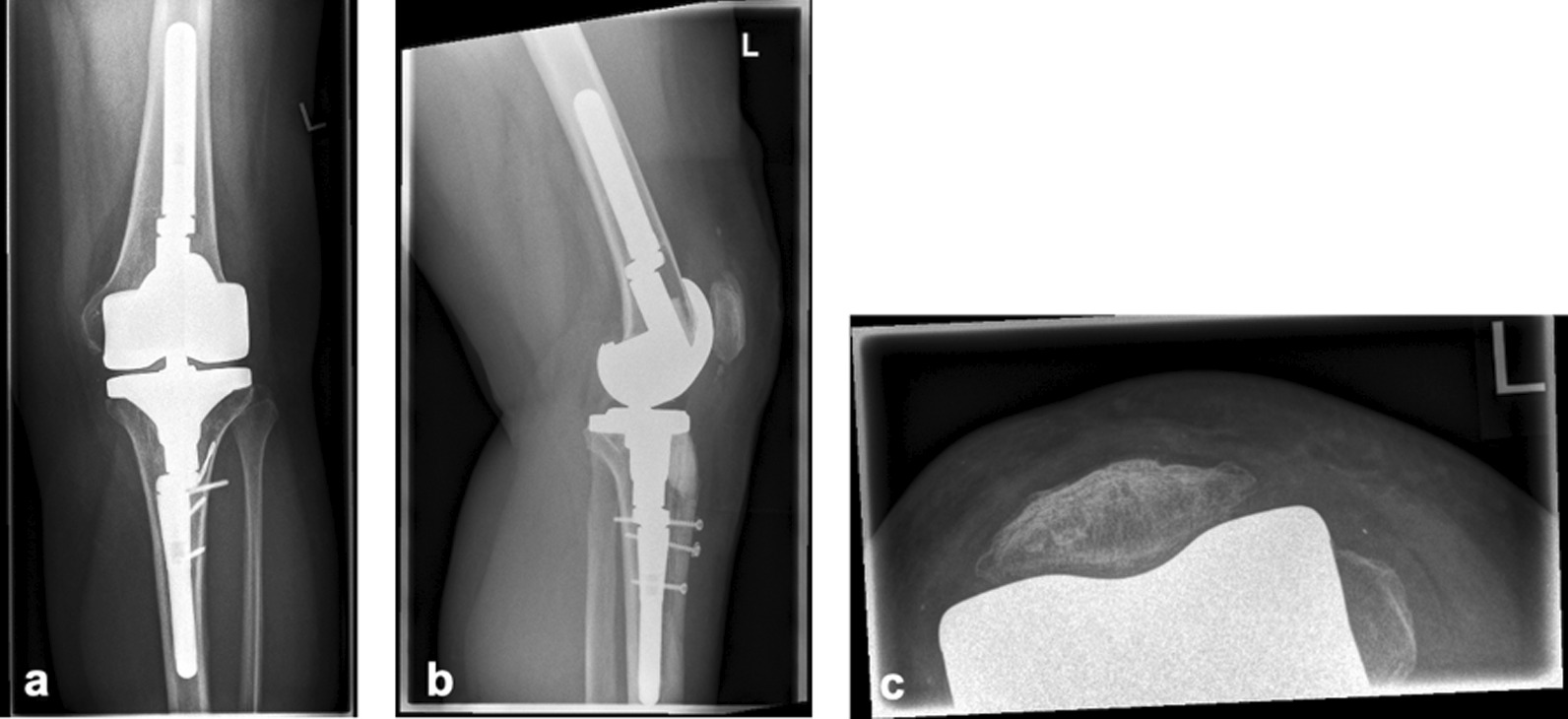
Radiologic follow up after ten month: X-ray shows correct implant position without periprosthetic bone lesions and centered patella tracking.** a** Frontal view.** b** Sagittal view shows still correct position of cement augmentation.** c** Axial view shows regular patella tracking

Follow-up was performed after 3 and 10 months. On clinical examination (Fig. 4), the patient showed irritant-free skin and soft tissue conditions; no redness or overheating; extension/flexion 0–0–110°; straight-leg raise completely possible; and force level 4/5 on side comparison of the knee stretchers. Active knee stretching with a flexed knee joint was possible without any problems. There was a centered patella run and no subluxation of the patella. Peripheral circulation, motor skills, and sensitivity were intact. X-ray confirmed correct implant position and central patella tracking (Fig. 5). The patient was highly pleased by the restored extension function and significantly reduced knee pain.

## Discussion

Periprosthetic tibial fractures are difficult to treat since the tibial segment hinders the fixation of osteosynthesis implants. A minimum of eight screws or the use of locking plates is recommended, but nevertheless, there are high rates of nonunion and dislocation of tibial fragments [5]. In the present case, we presented a surgical method to preserve extension functionality after Felix IV fracture and failed osteosynthesis of tibial tubercle fragment.

Multiple operation techniques exist to restore the knee joint’s extension system [6]. While primary repair was not possible since the necrotic tibial tubercle and concomitant patellar tendon insertion had to be removed, augmentation techniques were required. Not only autologous but also allograft reconstructions have been described resulting in unsatisfying long-term results with progressive extensor lag due to poor tissue quality [7–9]. Therefore, Browne *et al*. developed a synthetic augmentation technique to restore patellar tendon function providing long-term tensile strength and extensor functionality [10]. So, overall, in the case of patellar tendon or quadriceps tendon rupture, synthetic augmentation techniques are recommended [6]. Attachment tubes are commonly used in tumor surgery to restore function and stability after joint removal. Among them, reconstruction of the extension system of the knee joint in combination with a megaprosthesis is an approved method with promising outcomes concerning knee extension and knee flexion. On the one hand, in the case of megaprostheses, the attachment tube is fixed to the retention strings or a specific ventral anchorage block of the prosthetic device [11–14]. On the other hand, in the case of TKA with surrounding tibial bone, Browne *et al*. fixed the mesh graft intramedullary by bone cement instead of superficial anchorage to avoid proximal tibial fracture or tibial tubercle nonunion [10].

The present case required a different fixation technique since the tibial prosthesis was not designed to serve as a fixing point for the polyethylene terephthalate tube and the overlaying tibial tubercle as intramedullary anchorage had to be removed. Due to the extension strength acting on the tube and concomitantly on the attachment point, a direct fixation only on the ventral surface of the prosthesis would lead to ventral breakaway of the prosthesis aggravated by the missing frontal stability. Therefore, we compensated for the tibial defect by cement augmentation and attached the tube to the proximal tibia with two cancellous bone screws. As approved in tumor surgery [12], nonabsorbable sutures were used to fix the tube to the joint capsule and the extension system. Since the attachment tube in combination with endoprosthetic material is known to exhibit high infection rates [15], extensive jet lavage was performed before wound closure.

Within 6 months, the polyethylene terephthalate tube is known to be completely interspersed with fibroblasts resulting in a scarred tear-resistant tissue plate (16). So, when the tube stability reduces over time, its function will be replaced by autologous tissue to maintain tensile strength.

## Conclusion

In cases of Felix IV fractures, the use of plates and screws is recommended. Nevertheless, the management is often complicated by the underlying prosthesis components. We presented a reasonable surgical method using an attachment tube to treat dislocated Felix IV fractures with loss of tibial tubercle reducing knee pain and preserving extension functionality.

## Data Availability

Data sharing is not applicable to this article as no datasets were generated or analyzed during the current study.
